# WHO grade I meningiomas that show regrowth after gamma knife radiosurgery often show 1p36 loss

**DOI:** 10.1038/s41598-021-95956-x

**Published:** 2021-08-12

**Authors:** Pim J. J. Damen, Vincent J. Bulthuis, Patrick E. J. Hanssens, Suan Te Lie, Ruth Fleischeuer, Veerle Melotte, Kim A. Wouters, Andrea Ruland, Jan Beckervordersandforth, Ernst Jan M. Speel

**Affiliations:** 1 Department of Pathology, GROW School for Oncology and Developmental Biology, Maastricht University Medical Centre, P. Debyelaan 25, Postbox 5800, 6202 AZ Maastricht, The Netherlands; 2 Department of Neurosurgery, Maastricht University Medical Center, Maastricht, The Netherlands; 3 Gamma Knife Center Tilburg, ETZ-Elisabeth Hospital, Tilburg, The Netherlands; 4 Department of Pathology, ETZ-Elisabeth Hospital, Tilburg, The Netherlands

**Keywords:** Cancer, Molecular biology, Biomarkers

## Abstract

WHO grade I meningiomas occasionally show regrowth after radiosurgical treatment, which cannot be predicted by clinical features. There is increasing evidence that certain biomarkers are associated with regrowth of meningiomas. The aim of this retrospective study was to asses if these biomarkers could be of value to predict regrowth of WHO grade I meningiomas after additive radiosurgery. Forty-four patients with WHO grade I meningiomas who underwent additive radiosurgical treatment between 2002 and 2015 after Simpson IV resection were included in this study, of which 8 showed regrowth. Median follow-up time was 64 months (range 24–137 months). Tumors were analyzed for the proliferation marker Ki-67 by immunohistochemistry and for deletion of 1p36 by fluorescence in situ hybridization (FISH). Furthermore, genomic DNA was analyzed for promoter hypermethylation of the genes *NDRG1*–4, *SFRP1*, *HOXA9* and *MGMT*. Comparison of meningiomas with and without regrowth after radiosurgery revealed that loss of 1p36 (*p* = 0.001) and hypermethylation of *NDRG1* (*p* = 0.046) were correlated with regrowth free survival. Loss of 1p36 was the only parameter that was significantly associated with meningioma regrowth after multivariate analysis (*p* = 0.01). Assessment of 1p36 loss in tumor tissue prior to radiosurgery might be considered an indicator of prognosis/regrowth. However, this finding has to be validated in an independent larger set of tumors.

## Introduction

Meningiomas are brain tumors originating from the arachnoid cap cells of the arachnoid villi. They account for more than one third of all primary central nervous system neoplasms and are the second most common brain tumors^1^. Meningiomas are histologically classified according to the World Health Organization (WHO) into a benign, slowly growing WHO grade I (~ 80%) and the more invasive and aggressive atypical grade II (~ 18%) and anaplastic grade III (~ 2%) group^2^.

Generally, asymptomatic meningiomas are monitored regular follow up. When the tumor becomes symptomatic or shows radiological progression treatment can be indicated. The primary treatment of symptomatic meningiomas is surgery. Dependent on the completeness of resection, which can be indicated by Simpson grade I–IV, the 5-years recurrence free survival rate decreases from 98 to 57% respectively^3^. In patients with high surgical risk or tumors in eloquent or inaccessible regions either conventional or stereotactic radiotherapy is a viable alternative.^4^ Gamma Knife Radiosurgery (GKRS) is one of the most frequently used type of radiosurgery in meningiomas. The 5-year tumor control rate after GKRS is 95% in WHO grade I, 50% in grade II and 20% in grade III tumors^5^. Despite the fact that most WHO grade I meningiomas can be controlled well, a small group shows regrowth after radiosurgery, which is difficult to predict by histopathology on the tissue biopsy prior to treatment. One possibility would be that these tumors might present atypical morphological features which have been previously associated with a worse outcome^6^. Thus, it is crucial to analyse if such morphological features or other features such as molecular alterations can be found in WHO I tumors that show regrowth after radiosurgery.

Several studies have been conducted to investigate the molecular mechanisms underlying meningioma tumorigenesis, progression and recurrence. Some studies show that proliferation marker Ki-67 correlates with significantly shorter recurrence-free survival (RFS) in meningiomas of all grades, if for example tumors have a proliferation index > 3%^7^ or when > 25 positive cell nuclei per square millimetre^8^ are observed. Deletion of chromosome loci have been associated with meningioma recurrence, with 1p36 occurring most frequently. A study of 36 meningiomas (59% WHO 1) showed that tumors with 1p36 loss had a higher recurrence rate (25.9% vs. 11.5%, *p* = 0.003)^9^ and another study of 149 WHO 1 meningiomas showed that 1p36 loss correlated with a shorter RFS at 2.5, 5 and 10 years (*p* = 0.008, *p* = 0.009 and *p* = 0.01 respectively)^10^. The N-myc Downstream Regulated Gene (NDRG)-family has also been related to central nervous system (CNS)-tumors, including meningioma. Of the 4 family members, the *NDRG2* mRNA and protein expression levels were found to be reduced in higher grade and clinically aggressive meningiomas, which was associated with hypermethylation of the *NDRG2* promotor^11^.

In addition, a tree-fold reduction in *NDRG2* mRNA levels was reported when comparing primary with recurrent tumors (*p* = 0.009)^12^. In contrast, *NDRG4* has been shown to be overexpressed in high grade malignant meningioma cell lines. Although it is unknown how this overexpression is induced, silencing of *NDRG4* inhibited proliferation and induced apoptotic cell death^13,14^. A couple of other studies have reported silencing of gene expression by promotor hypermethylation to play a role in meningioma tumorigenesis. In a mRNA profiling study *SFRP1* was one of the genes which expression was downregulated in recurrent meningiomas (*p* = 0.02)^15,16^. In a genome-wide methylation analysis *HOXA9* was correlated with recurrence^17^. This was also found in an independent study analysing *HOXA7*, *9* and *10* (*p* = 0.0019)^18^. Finally, promotor hypermethylation of O^6^–methylguanine-DNA methyltransferase (*MGMT)* has been found in 0–22% in meningiomas and grade II meningiomas showed twofold higher percentage of MGMT promotor methylation than grade I tumor^19–21^.

The objective of this study was to investigate if the above-mentioned markers also can be used to recognise WHO grade I meningiomas that show regrowth after GKRS. Treatment for these potentially more aggressive tumors could be adapted.

For this purpose, 44 WHO grade I meningiomas, including 8 showing regrowth after radiosurgery, were analysed for Ki-67 and *NDRG4* protein expression, loss of 1p36 and promotor hypermethylation of the genes *NDRG1-4*, *SFRP1*, *HOXA9* and *MGMT*.

## Material and methods

A detailed description of materials and methods can be found in Supplementary “Methods”.

### Patient and tissue samples

Samples were collected from a series of ~ 600 meningiomas treated in ETZ-Elisabeth Hospital (Tilburg, Netherlands) with GKRS between 2002 and 2015. For this study we selected all WHO grade I meningiomas which were partially resected (Simpson grade IV; n = 44) and received additive GKRS. The median time between resection and additive GKRS was 7 months (range 1–13 months), with no proven regrowth within this period of time. Of the 44 meningiomas included, 8 tumors showed regrowth.

Tumor material collected at primary surgery was retrieved from 5 different Dutch hospitals. Patients were followed with a median follow-up of 64 months (range 24–137 months; regrowth group: 50 months [range 29–81 months]; no regrowth group: 73 months [range 23–137 months]). All regrowth was in-field. Histologically, 6 meningioma subgroups were classified. Table 1 summarizes clinical and histopathological parameters of the 44 meningiomas.

**Table 1:** Clinical, radiobiological and histopathological parameters in relation to regrowth of irradiated WHO grade I meningiomas.

	No regrowth	Regrowth	Total	Fisher’s exact (*p* value)	Kaplan–Meier (*p* value)
**No of patients**	36	8	44		
**Age (yrs)**				0.26	0.124
< 58	22 (88%)	3 (12%)	25		
≥ 58	14 (74%)	5 (26%)	19		
**Gender**				0.67	0.161
Female	26 (84%)	5 (16%)	31		
Male	10 (77%)	3 (23%)	13		
**Tumor localization**				0.11	0.056
Inside skull base	17 (94%)	1 (6%)	18		
*(Petro)Clival*	*2*	*0*	*2*		
*Cavernous Sinus*	*4*	*0*	*4*		
*Frontobasal*	*1*	*0*	*1*		
*Parasellar*	*2*	*0*	*2*		
*Petrous Bone*	*2*	*0*	*2*		
*Planum Sphenoidale*	*1*	*0*	*1*		
*Sella*	*0*	*1*	*1*		
*Sphenoid Wing*	*1*	*0*	*1*		
*Cerebral Pontine Angel*	*4*	*0*	*4*		
Outside skull base	19 (73%)	7 (27%)	26		
*Convexity*	*4*	*2*	*6*		
*Falcine*	*5*	*2*	*7*		
*Orbital*	*1*	*0*	*1*		
*Parasagital*	*0*	*1*	*1*		
*Posterior Fossa*	*3*	*1*	*4*		
*Tentorial*	*5*	*0*	*5*		
*Tentorial Notch*	*1*	*1*	*2*		
**Tumor volume (cm**^**3**^)				0.69**	0.559
0 to 5	23	4	27		
5 to 10	9	2	11		
10 to 15	4	2	6		
**Dose 100%**				1	0.663
< 11 Gy	9 (82%)	2 (18%)	11		
≥ 11 Gy	27 (82%)	6 (18%)	33		
**Prescribed dose**				1	0.565
< 13 Gy	8 (80%)	2 (20%)	10		
≥ 13 Gy	28 (82%)	6 (18%)	34		
**Histotype**				0.45	0.211
Meningothelial	21 (78%)	6 (22%)	27		
Not meningothelial	15 (88%)	2 (12%)	17		
*Fibrous*	*4*	*0*	*4*		
*Transitional*	*8*	*1*	*9*		
*Angiomatous*	*1*	*0*	*1*		
*Psammomatous*	*2*	*0*	*2*		
*Microcystic*	*0*	*1*	*1*		

**After combining tumor volume groups ‘5–10 cm^3^’ and ’10–15 cm^3^’.

This study was approved by the Medical Ethical Committee (METC 154-098, MUMC Maastricht). Patient material was used according to the Code for Proper Secondary Use of Human Tissue (Federation of Medical Scientific Societies, The Netherlands; 2013).

### Radiotherapy

Treatment planning for single session GKRS using Leksell Gamma Knife 4C or Perfexion (Elekta AB, Stockholm, Sweden) was performed with Leksell Gamma Plan (Elekta AB) based on high resolution Gadolinium enhanced stereotactic planning T1-weighted MRI scans with G-frame. The target volume was defined as the contrast-enhancing lesion. Only part of the dural tail adjacent to the tumor was included in the target volume planning^22^. A median dose of 11 Gy (10–18 Gy) was prescribed to that isodose-line covering 90–100% of the target volume, resulting in a median marginal dose of 11 Gy (10–17.8 Gy).

Follow-up imaging was carried out at 6 months after radiosurgery, followed by a 1-year interval in the next 3 years. Tumor growth was defined as a minimum diameter increase of 2 mm in any direction. Tumor growth was considered an out-field increase of tumor volume in the axial planes. If growth was observed inside the treated volume it was classified as in-field regrowth, whereas growth outside the treatment volume was considered out-field regrowth.

### Immunohistochemistry

Immunohistochemistry on 3 µm-thick formalin-fixed, paraffin-embedded (FFPE) tissue sections using primary antibodies directed against Ki-67 (MIB-1) and NDRG4, was performed using the DAKO Autostainer Link 48, according to standard protocols. MIB-1 nuclear staining was subdivided in 2 categories: ≤ 1% and > 1%. For *NDRG4* staining the intensity (0–3 +) and percentage for both nuclear and/or cytoplasmic stained tumor cells were evaluated.

### Fluorescence in situ hybridization (FISH)

FISH was performed on 3 µm-thick FFPE tissue sections, using a 1p36/1q25 specific probe mixture, as previously described^23^. At least 20 non-overlapping nuclei per sample were scored for evaluation, using a Leica DM5000b fluorescence microscope, with appropriate fluorochrome filter sets. 1p36 deletion was indicated if the ratio between 1p36/1q25 was less than 0.8.

### DNA isolation from tumor tissue

DNA was isolated using the Promega Maxwell 16 FFPE plus LEV DNA purification kit according to manufacturer’s instructions.

### Methylation-specific polymerase chain reaction (MSP)

The methylation status of *NDRG1-4, MGMT, SFRP1, HOXA9 and MGMT* was assessed by MSP. 500 ng DNA was modified by sodium bisulphite, using the Zymo Research EZ DNA Methylation kit, according to manufacturer’s instructions. Primer sequences and corresponding annealing temperatures are summarised in Supplementary Table S1. The PCR products were analysed on a 2% agarose gel.

### Statistical analysis

Statistical analysis was processed with the statistical package for the social sciences (SPSS, version 23 Chicago, IL) computer software for Windows. Fisher’s Exact test was used for correlating parameters with regrowth. Kaplan–Meier’s Log-Rank test was used for univariate analysis of progression free survival. Multivariate analysis was performed using Cox Regression Model. Statistical significance was presumed as *p* < 0.05.

### Ethics approval and consent to participate

This study was approved by the Medical Ethical Committee (METC 15-4-098, MUMC Maastricht). Patient material was used according to the Code for Proper Secondary Use of Human Tissue (Federation of Medical Scientific Societies, The Netherlands; 2013).

### Conference presentation

This study was presented in part orally at the Leksell Gamma Knife^®^ Society Meeting, Amsterdam, May 15–19, 2016.^24^

## Results

A total of 44 meningiomas from 44 patients were included in this study, of which 8 (18%) showed regrowth during follow-up after a median time of 51 months. Local control rate was 100% after 2 years and 89% after 5 years. In all cases, the regrowth occurred infield and no marginal regrowth was observed. Clinical, radiobiological and histopathological parameters, including tumor volume and radiotherapy dose, in relation to regrowth can be found in Table 1. These parameters did not show significant association with regrowth.

**Figure 1:**
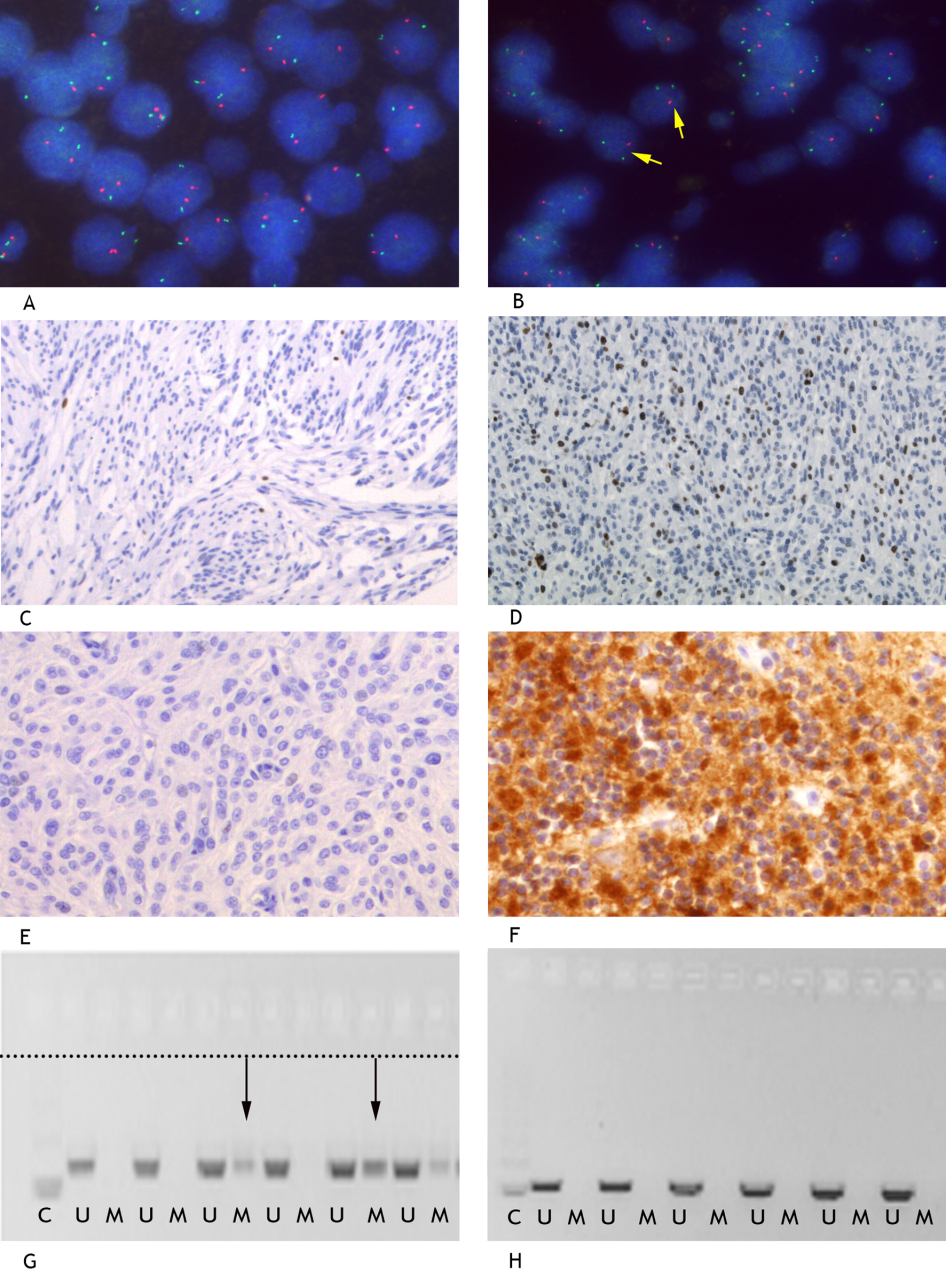
Representative examples of molecular analysis of WHO grade I meningiomas. (**A**-**B**): FISH analysis showing in (**A**) a tumor with two copies of 1p36 (red) and 1q25 (green) in DAPI counterstained tumor cell nuclei (blue). This tumor did not show regrowth after irradiation. In (**B**) arrows point to nuclei showing only one copy of 1p36 (red), indicating 1p36 loss. This tumor showed regrowth after irradiation. (**C**-**F**): Immunohistochemical analysis of Ki-67 (**C**-**D**) and NDRG4 (**E**–**F**) showing in (**C**) a tumor with Ki-67 expression < 1.0%, (**D**) a tumor with Ki-67 expression > 1.0%, (**E**) a tumor with no NDRG4 expression and in (**F**) a normal brain tissue control with positive NDRG4 expression. (**G**-**H**): Cropped MSP analysis of NDRG1 (**G**) and NDRG4 (**H**) (full blots in Supplementary Figs. S1-2). In (**G**) 2 of 6 tumors show NDRG1 hypermethylation, dashed line separates upper and lower side of the gel; compiled from 2 photographs of the gel, whereas no tumors show NDRG4 hypermethylation (**H**). Abbreviations: U = unmethylated; M = methylated. C = unmethylated control DNA (normal lymphocytes). Magnification 100 × (**C**-**D**), 200 × (**E**–**F**), 400 × (**A**-**B**).

**Figure 2:**
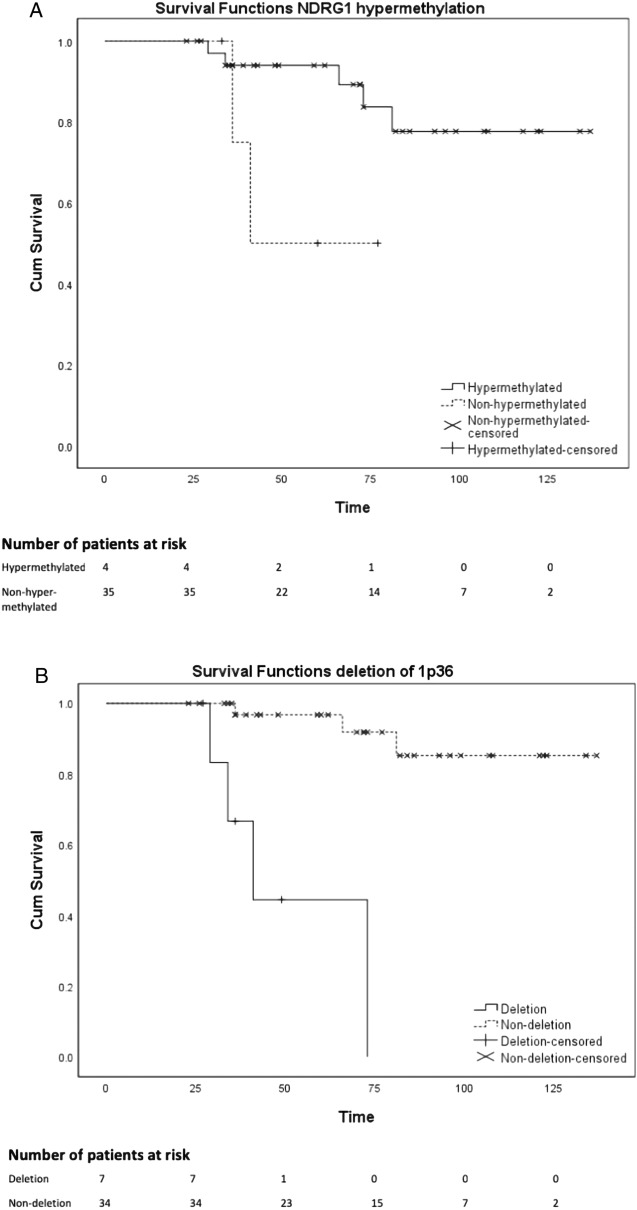
Kaplan Meier survival functions of (**A**) NDRG1 hypermethylation and (**B**) deletion of 1p36 in relation to meningioma regrowth.

FISH for 1p36/1q25 was performed on 43 meningioma samples and 2 nontumoral brain tissue control samples (Fig. 1A-B). One meningioma sample did not have enough tissue for FISH analysis. Deletion of chromosome 1p36 was detected in 8 meningiomas. The nontumoral brain tissue control samples showed disomy for both 1p36 and 1q25. Five out of 8 (63%) meningiomas with chromosome 1p36 deletion showed regrowth, compared to 3 out of 32 (9%) samples without deletion of 1p36 (*p* = 0.003). Chromosome 1p36 loss was correlated with regrowth (*p* = 0.003) as well as regrowth free survival (*p* = 0.001), as seen in Table 2 and Fig. 2.

**Table 2:** Molecular parameters in relation to regrowth of irradiated WHO grade I meningiomas.

	No regrowth	Regrowth	Total	Fisher’s Exact (p value)	Kaplan–Meier (p value)
**Ki-67 Expression**				1	0.207
≤ 1.0%	19 (83%)	4 (17%)	23		
> 1.0%	17 (81%)	4 (19%)	21		
**Deletion 1p36**				0.003*	0.001*
Yes	3 (38%)	5 (63%)	8		
No	32 (91%)	3 (9%)	35		
***NDRG1***				0.20	0.046*
No methylation	31 (86%)	5 (14%)	36		
Methylation	3 (60%)	2 (40%)	5		
***NDRG2***				0.20	0.31
No methylation	31 (86%)	5 (14%)	36		
Methylation	3 (60%)	2 (40%)	5		
***NDRG3***				-	-
No methylation	34 (83%)	7 (17%)	41		
Methylation	0	0	0		
***NDRG4***				-	-
No methylation	34 (83%)	7 (17%)	41		
Methylation	0	0	0		
***SFRP1***				1	0.67
No methylation	28 (82%)	6 (18%)	34		
Methylation	6 (86%)	1 (14%)	7		
***HOXA9***				1	0.40
No methylation	8 (89%)	1 (11%)	9		
Methylation	26 (81%)	6 (19%)	32		
***MGMT***				0.69	0.87
No methylation	22 (85%)	4 (15%)	26		
Methylation	12 (80%)	3 (20%)	15		

* Statistical significance was presumed as *p* < 0.05.

Nuclear Ki-67 expression was examined in 44 meningioma samples and 2 nontumoral brain tissue control samples using MIB-1 immunostaining (Fig. 1C-D). Twenty-three meningiomas had a proliferation index ≤ 1.0%, of which 4 (17%) showed regrowth, compared to 19 (83%) showing no regrowth. Twenty-one meningiomas had labelling > 1.0%, of which 4 (19%) showed regrowth, compared to 17 (81%) non-regrowth. Ki-67 proliferation index did not correlate with survival.

MSP for *NDRG1-4*, *SFRP1*, *HOXA9* and *MGMT* was performed on 41 meningioma samples (see Table 2 and Fig. 1G-H) and 10 nontumoral control brain tissue samples. From 3 meningioma samples too less DNA could be isolated for MSP analysis. No hypermethylation was found for *NDRG3* and *NDRG4,* and *NDRG4* protein expression could not be detected by immunohistochemistry (Fig. 1E–H). The promotor regions of *NDRG1* and *2* were hypermethylated in 5 samples of which 40% showed regrowth, compared to 14% in the non-hypermethylated group. For *MGMT* this was 20% vs. 15%, for *HOXA9* 18% vs. 11% and for *SFRP1* 14% vs. 18%, respectively. None of 10 nontumoral control samples showed hypermethylation for any of the genes analysed. Only hypermethylation of *NDRG1* was correlated with regrowth free survival (*p* = 0.046).

Multivariate Cox Regression analysis was performed for all parameters which were significant (p < 0.05) on univariate analysis of regrowth free survival (i.e. deletion 1p36 and methylation of *NDRG1*). Deletion of 1p36 kept its significance after multivariate analysis (*p* = 0.01).

## Discussion

WHO grade I meningioma patients who undergo additive radiosurgical treatment after Simpson grade IV resection sometimes show tumor regrowth. The aim of this retrospective study was to asses if biomarkers may be of value to predict regrowth of WHO grade I meningiomas after additive radiosurgery. Treatment for these potentially more aggressive tumors then could be adapted.

From a series of 44 of these cases, treated in a Dutch GKRS center in the period of 2002–2015, 8 patients showed regrowth. We found loss of chromosome 1p36 and to a lesser extent hypermethylation of *NDRG1* to be correlated with regrowth free survival, while clinical, radiobiological and histopathological parameters, expression of Ki-67 and *NDRG4* and hypermethylation of *NDRG2*-4, *SFRP1*, *HOXA9* and *MGMT* were not.

Chromosome 1p loss is the second most common abnormality in meningioma and is more frequently detected in higher grade tumors. Loss of 1p has been found in 13–26% of Grade I, 40–76% of Grade II and 70–100% of Grade III meningiomas^25–27^. Loss of 1p, along with 9p and 14q, have been identified more often in high grade meningiomas which show higher rate of recurrence^28^. Interestingly, Grade I meningiomas showing recurrence had significantly more chromosome copy number alterations with a mean of 19% of genome disrupted, including 1p deletion, compared to 3% in low-grade samples not showing recurrence^29^. Tabernero et al. found that the common deleted region of 1p was pter → 1p34.2^30^, which contains the 1p36 locus evaluated in our present study. A gene mapped in this region is the ALPL gene (1p36.12-34), coding for the alkaline phosphatase enzyme. In a study of 66 meningiomas loss of 1p was strongly correlated with a reduced activity of the alkaline phosphatase enzyme^31^. An additional study of 54 meningiomas showed that decreased expression of alkaline phosphatase was correlated with recurrence (*p* = 0.0064) and shorter RFS (*p* = 0.035).^32^.

Although so far no chromosome 1p methylation data have been reported, some studies have examined hypermethylation of gene promotors, for example that of TP73. Hypermethylation of TP73 has been found more frequently in meningiomas with chromosome 1p loss^33^. TP73 is suggested as potential biomarker for higher grade meningiomas, because 70–80% of high-grade tumors have TP73 promotor hypermethylation. Since the TP73 gene is included in the 1p36 Vysis FISH probe applied in the present study, it is tempting to speculate that this biomarker may also be of value to predict meningioma regrowth, which remains to be studied^28^. Mutations in well-known cancer genes located on chromosome 1p, such as *NRAS* (1p13.2), *GADD45A* (1p31.2–31.1), *CDKN2C* (1p32.2), *RAD45*(1p32) and *EPB41* (1p36.2–p34), have so far only sporadically been detected^26,28,34,35^.

Another interesting finding in our study is that *NDRG1* hypermethylation in meningiomas with regrowth was associated with unfavorable regrowth-free survival. The NDRG family consists of four different members, namely *NDRG1*, *2*, *3*, and *4*. This family of genes is involved in the regulation of cell proliferation and differentiation, and all members have specific functions^36^. Hypermethylation of *NDRG1* has been reported to promote pathogenesis and/or proliferation of prostate, breast and gastric cancer^37^. A tumor suppressor function for NDRG1 has also been implicated in CNS-tumors, including neuroblastoma^38^ and glioma^39^. To our knowledge our study is the first to correlate *NDRG1* hypermethylation with meningioma regrowth after radiotherapy, which remains to be further studied. We could not provide evidence for *NDRG2* hypermethylation as was reported by Lusis et al. as a mechanism of *NDRG2* downregulation in relation to tumor recurrence^11^. *NDRG4* is usually expressed in heart and brain cells, and has been recently found to be downregulated in colorectal cancer as a result of promotor hypermethylation^40^. In contrast, Kotipatruni et al. showed that *NDRG4* was overexpressed in the high grade meningioma cell lines IOMM-Lee and CH-157 MN^13^. In a subsequent study, these authors demonstrated that *NDRG4* silencing inhibits proliferation and induces apoptosis in these cell lines^14^, suggesting an oncogenic role for *NDRG4* in meningiomas. In our study *NDRG4*, highly expressed in all nontumoral brain control tissue, was not expressed in WHO I meningiomas. Moreover, we could not demonstrate that *NDRG4* promotor hypermethylation is the possible mechanism downregulating *NDRG4* expression in low grade meningiomas. A potential role for *NDRG3* hypermethylation within nervous system malignancies remains uncertain as also in this study no *NDRG3* promotor hypermethylation could be detected.

Although hypermethylation of *MGMT* did not correlate with regrowth, 35% of meningioma grade I patients showed hypermethylation of *MGMT*, which is relatively high in comparison with 0–22% in earlier reports^19–21^. However, *MGMT* hypermethylation does not appear to be of value as an indicator for temozolomide chemotherapy in the treatment of meningiomas, because so far temozolomide has been found ineffective^41^.

In the current study, not all parameters related to meningioma progression have been addressed^1^. For example, TERT promotor mutations have been found significantly associated with recurrence with or without progression to a higher grade^42,43^. In this respect we could analyze 6 of our regrowth cases by digital droplet PCR which did not harbor a mutation at TERT promotor hotspot regions C228 and C250 of TERT. As a result it is unlikely that TERT promotor mutations have value in the prediction of meningioma WHO grade I regrowth after GKRS in the current study.

In contrast to some molecular markers, we did not identify clinical or radiobiological factors associated with tumor regrowth, as shown in Table 1. Dibiase et al. analyzed 139 benign meningiomas treated with GKRS and found gross tumor volume (GTV) > 10 cc significantly correlated with unfavorable 5-year disease-free and overall survival (*p* = 0.038 and *p* = 0.0001, respectively)^44^. We did not find a correlation between tumor volume and regrowth. The difference with our study might be explained by the fact that only 38% of their patients were treated post-operatively and no histopathologic proof of benign (WHO grade I) disease was available. Furthermore, we did not find a correlation between radiotherapy dose and regrowth. Kuhn et al. showed in a study of 279 meningiomas (21% WHO grade I) treated with GKRS marginal doses of ≥ 12 Gy associated with improved local control (*p* = 0.015)^45^. The difference with our study could be explained by the fact they included a less uniform group, with 20% multifocal disease, 61% unknown histology and only 43% of tumors treated with GKRS post-operatively. Besides, 63% of their local failures was classified as marginal, whereas we did not have marginal regrowth, but only in-field.

When there would be enough evidence to predict regrowth in WHO grade I meningiomas with a biomarker, the question arises how one should treat these potentially more aggressive tumors. To the best of our knowledge no studies have investigated this research question yet. One option could be to consider these tumors as progressive disease and a logical approach then would be to opt for a more frequent follow-up schedule and/or dose escalation.

Although we have studied a uniformly postoperatively GKRS treated group of WHO grade I meningiomas, our series consisting of 44 tumors, including 8 regrowth cases, may be considered relatively small. This impacts the statistical analysis, therefore this study should be regarded as hypothesis generating. Another limitation is the relatively short follow-up time for some patients, ranging from 24 to 137 months, and the median time to regrowth in this study being 51 months. It therefore might be possible that some regrowth cases have been missed. Additional limitations of this study are its retrospective character and the fact that only Gamma Knife irradiated patients were included, whereas there are other radiotherapy modalities applied in the treatment of meningioma patients.

## Conclusions

In conclusion, our results indicate that loss of 1p36 is associated with regrowth after GKRS of WHO grade I meningiomas, which might have implications for treatment. In order to consider 1p36 deletion as a putative indicator for regrowth, our findings need to be validated in an independent larger set of tumors.

## Supplementary Information

Supplementary Information.

## Data Availability

All data generated or analyzed during this study are included in this published article and its supplementary information files.
