# Supplementary material for: WHO grade I meningiomas that show regrowth after gamma knife radiosurgery often show 1p36 loss
Source: Sci Rep. 2021 Aug 12;11:16432. doi: 10.1038/s41598-021-95956-x (PMC8361078; doi:10.1038/s41598-021-95956-x)
Supplement: Supplementary file 1 — Supplementary Information. [file 41598_2021_95956_MOESM1_ESM.docx]

**Supplementary Files**

**Title:** WHO Grade I Meningiomas that show regrowth after Gamma Knife Radiosurgery often show 1p36 loss

**Authors:** Pim J.J. Damen, MD^1†^, Vincent J. Bulthuis, MD^2†^, Patrick E.J. Hanssens, MD, PhD^3^, Suan Te Lie, MD, PhD^3^, Ruth Fleischeuer, MD^4^, Veerle Melotte, PhD^1^, Kim A. Wouters^1^, Andrea Ruland^1^, Jan Beckervordersandforth, MD, PhD^1^, Ernst Jan M. Speel, MD, PhD^1*^

^†^: Authors contributed equally

*: Corresponding Author

**Affiliations**:

1. Department of Pathology, GROW school for Oncology & Developmental Biology, Maastricht University Medical Centre, Maastricht, the Netherlands

2. Department of Neurosurgery, Maastricht University Medical Center, Maastricht, The Netherlands

3. Gamma Knife Center Tilburg, ETZ-Elisabeth Hospital, Tilburg, The Netherlands

4. Department of Pathology, ETZ-Elisabeth Hospital, Tilburg, The Netherlands

**Corresponding author:**

Prof. dr. E.J.M. Speel

Department of Pathology

P. Debyelaan 25

Postbox 5800 6202 AZ, Maastricht

T: +31 (0)43 3874610

E-mail: ernstjan.speel@mumc.nl

**Supplementary Materials and Methods**

*Patient and tissue samples:*

Samples were collected from a series of ~600 meningiomas treated in ETZ-Elisabeth Hospital (Tilburg, Netherlands) with GKRS between 2002-2015. For this study we selected all WHO grade I meningiomas which were partially resected (Simpson grade IV; n=44) and received additive GKRS. The median time between resection and additive GKRS was 7 months (range 1-13 months), with no proven regrowth within this period of time. Of the 44 meningiomas included, 8 tumors showed regrowth.

Tumor material collected at primary surgery was retrieved from 5 different Dutch hospitals: ETZ-Elisabeth, Canisius Wilhelmina (Nijmegen), Radboud UMC (Nijmegen), Zuyderland (Heerlen) and UMC Groningen. Patients were followed with a median follow-up of 64 months (range: 24 – 137 months). All regrowth was in-field. Histologically, 6 meningioma subgroups were classified. Table 1 summarizes clinical and histopathological parameters of the 44 meningiomas.

This study was approved by the Medical Ethical Committee (METC 15-4-098, MUMC Maastricht). Patient material was used according to the Code for Proper Secondary Use of Human Tissue (Federation of Medical Scientific Societies, The Netherlands; 2013).

*Radiotherapy:*

Treatment planning for single session GKRS using Leksell Gamma Knife 4C or Perfexion (Elekta AB, Stockholm, Sweden) was performed with Leksell Gamma Plan (Elekta AB) based on high resolution Gadolinium enhanced stereotactic planning T1-weighted MRI scans with G-frame. The target volume was defined as the contrast-enhancing lesion. Only part of the dural tail adjacent to the tumor was included in the target volume planning.[24] A median dose of 11 Gy (10-18 Gy) was prescribed to that isodose-line covering 90-100% of the target volume, resulting in a median marginal dose of 11 Gy (10-17.8 Gy).

Follow-up imaging was carried out at 6 months after radiosurgery, followed by a 1-year interval in the next 3 years. Tumor growth was defined as an out-field increase of tumor volume in the axial planes. If progression was observed inside the treated volume it was classified as in-field progression.

*Immunohistochemistry*

Immunohistochemistry for Ki-67 and NDRG4 was performed on 3µm sections with a Dako Autostainer Link 48 [Agilent Technologies Inc., Santa Clara, California, USA]. Antigen retrieval for Ki-67 staining was performed in FLEX high TRS (DAKO EnVision™ FLEX Target Retrieval Solution, pH 9.0, 95 °C, 15 min) and for NDRG4 staining in FLEX low TRS (pH 6.0, 95 °, 15 min). The staining procedure included incubation in peroxidase blocking solution (FLEX Peroxidase-Blocking Reagent), incubation with primary antibody Ki-67 (clone MIB-1 dilution 1/50; DAKO, Amstelveen, The Netherlands) or NDRG4 (clone D4A6; dilution 1/50; Cell Signaling, Leiden, The Netherlands), incubation in detection reagent (Dako EnVision™ FLEX/HRP Detection Reagent) and finally in a substrate-chromogen solution (DAKO FLEX DAB+ Chromogen, Substrate Buffer).

MIB-1 staining was evaluated semi-automatically using the Leica QWin V3 [Leica Microsystems, Wetzlar, Germany] and expressed as percentage positive tumor cells (proliferation index). A minimum of 1000 nuclei were counted from 5 microscope fields (x200) containing the highest density of positive tumor cells. Immunostaining was subdivided in 2 categories: ≤1% and >1%. For *NDRG4* staining the intensity (0–3+) and percentage for both nuclear and/or cytoplasmic stained tumor cells were evaluated.

*Fluorescence in situ hybridization (FISH)*

FISH was performed on 3µm-thick formalin-fixed, paraffin-embedded (FFPE) tissue sections. Slides were baked at 65ºC for 30 min, deparaffinized three times in Xylene and two times in 100% ethanol for 3 min, airdried and incubated for 20 min in 0.2M HCL at RT. After washes in both distilled water and 2xSSC for 3 min each slides were incubated in 1M NaSCN at 80ºC for 30 min, washed in distilled water and 2xSSC, and incubated in 100ml protease solution (NaCl 8 g/l, pH 2.0) with 100mg pepsin at 37ºC for 20 min. The slides were washed in 2xSSC and incubated in 4% formalin at RT for 10 min. After washing in 2xSSC and distilled water, tissue was dehydrated in an alcohol series and air dried. Six microliter 1p36/1q25 probe mixture (Vysis LSI 1p36/LSI 1q25 Dual-Color Probe; Abbott Molecular, Abbott Park, Illinois, U.S.A.) was pipetted on the slides. Probe and target DNA were denatured at 85ºC for 5 min and incubated overnight at 37ºC in a humidified chamber. Slides were washed in 2xSSC/0,3% NP-40 three times: at RT for 5 min, at 73ºC for two min (stringency wash) and at RT for 1 min. Slides were dehydrated and counterstained with 0.2µg 4’,6-diamidino-2-phenylindole dihydrochloride (DAPI) per mililiter Vectashield antifade solution [Vector Laboratories, Burlingame, California, USA]. At least 20 non-overlapping nuclei per sample were scored for evaluation, using a Leica DM5000b fluorescence microscope, with appropriate fluorochrome filter sets. 1p36 deletion was indicated if the ratio between 1p36/1q25 was less than 0.8.

*DNA isolation from tumor tissue*

DNA was isolated using the Maxwell 16 FFPE plus LEV DNA purification kit [Promega, Fitchburg, Wisconsin, USA] according to manufacturer’s instructions. Briefly, five 8µm FFPE slides of meningioma tissue were added to 180µl Incubation Buffer and 20µl proteinase K. Overnight incubation was performed at 70ºC [Eppendorf thermomixer, Nijmegen, The Netherlands] at 800rpm. After adding 400µl lysis buffer, samples were run in a Maxwell 16 MDx [Promega] to isolate DNA. DNA concentration and purity were measured using a Quantus spectrophotometer [Promega]. DNA was stored at 4ºC.

*Methylation-specific polymerase chain reaction (MSP)*

Using MSP assay, the methylation status of *NDRG1-4, MGMT, SFRP1, HOXA9 and MGMT* was assessed. 500ng DNA was modified by sodium bisulphite, using the EZ DNA Methylation kit [Zymo Research, Irvine, California, USA], according to manufacturer’s instructions. Completely methylated control DNA (in vitro methylated DNA) was used as positive control, completely unmethylated control DNA (normal lymphocytes) was used as negative control, and water served as a blanc. MSP assay was carried out in a T100™ Thermal cycler [Bio-Rad, Hercules, California, USA] or a Veriti 96 well thermal cycler [Thermo Fisher Scientific, Franklin, Massachusetts, USA]. Primer sequences and corresponding annealing temperatures are summarised in table 2. The PCR products were analysed on a 2% agarose gel.

*Statistical analysis*

Statistical analysis was processed with the statistical package for the social sciences (SPSS, version 23 Chicago, IL) computer software for Windows. Fisher’s Exact test was used for correlating parameters with regrowth. Kaplan-Meier’s Log-Rank test was used for univariate analysis of progression free survival. Multivariate analysis was performed using Cox Regression Model. Statistical significance was presumed as p<0.05.

**Supplementary table S1: Primer sequences and corresponding annealing temperatures**

| **Gene symbol** | **Primer*** | **Primer sequence (5'-3')** | **Product size (bp)** | **Primer annealing temperature (˚C)** | **Cycli** |
| --- | --- | --- | --- | --- | --- |
| ***NDRG1*** | FU | GTT ATT TTY GTT TTY GTT TAT TTT TT | 148 | 56 | 35 |
|  | FD | TCC CRA AAC TAA ATC AAA AAC C | 148 | 56 | 35 |
|  | US | GTT ATT TTT GTT TTT GTT TAT TTT TTT TT | 145 | 64 | 35 |
|  | UaS | CAA AAC TAA ATC AAA AAC CAC AAC A | 145 | 64 | 35 |
|  | MS | TTT TCG TTT TCG TTT ATT TTT TTT C | 137 | 64 | 35 |
|  | MaS | ACT AAA TCA AAA ACC GCG ACG | 137 | 64 | 35 |
| ***NDRG2*** | FU | YGT TTT TTA TTT ATA GYG GTT TTT | 125 | 56 | 35 |
|  | FD | TCC TAA TAC CTC TCC TCT CTT TAC TAC | 125 | 56 | 35 |
|  | US | TTT TAT TTA TAG TGG TTT TTT GTA TTT TTT | 111 | 62 | 35 |
|  | UaS | TCT CCT CTC TTT ACT ACA TCC CAA CA | 111 | 62 | 35 |
|  | MS | TTT ATA GCG GTT TTT CGT ATT TTT C | 103 | 64 | 35 |
|  | MaS | CCT CTC TTT ACT ACG TCC CGA CG | 103 | 64 | 35 |
| ***NDRG3*** | FU | YGT TTT AGG TGA GTG AGY GTT TT | 90 | 56 | 35 |
|  | FD | AAC ACR AAC CAC CAA AAC CTC | 90 | 56 | 35 |
|  | US | GTT TTA GGT GAG TGA GTG TTT TTG TTT | 78 | 68 | 30 |
|  | UaS | AAA CAA AAA AAC AAC ACC CCC A | 78 | 68 | 30 |
|  | MS | TAG GTG AGT GAG CGT TTT CGT TC | 69 | 68 | 30 |
|  | MaS | AAA AAA CGA CGC CCC CG | 69 | 68 | 30 |
| ***NDRG4*** | FU | ATY GGG GTG TTT TTT AGG TTT | 138 | 56 | 35 |
|  | FD | ATA CCR AAC CTA AAA CTA ATC CC | 18 | 56 | 35 |
|  | US | GGG TGT TTT TTA GGT TTC GCG TCG C | 126 | 66 | 30 |
|  | UaS | CCT AAA ACT AAT CCC AAA CAA ACC A | 128 | 66 | 30 |
|  | MS | TTT TTT AGG TTT CGC GTC GC | 117 | 66 | 30 |
|  | MaS | AAA CTA ATC CCG AAC GAA CCG | 117 | 66 | 30 |
| ***SFRP1*** | FU | TTT AGT TTT GTA GTT TTY GGA GTT AG | 157 | 56 | 35 |
|  | FD | CCC CRA CCA ATA ACR ACC CTC | 157 | 56 | 35 |
|  | US | GTT TTG TAG TTT TTG GAG TTA GTG TTG TGT | 135 | 66 | 25 |
|  | UaS | CTC AAC CTA CAA TCA AAA ACA ACA CAA ACA | 135 | 66 | 25 |
|  | MS | TGT AGT TTT CGG AGT TAG TGT CGC GC | 126 | 66 | 25 |
|  | MaS | CCT ACG ATC GAA AAC GAC GCG AAC G | 126 | 66 | 25 |
| ***HOXA9*** | FU | ATG ATT GTA AAA TAT YGG ATT ATT AAT AG | 132 | 56 | 35 |
|  | FD | AAA AAT ACA ATC ACC TAA TAA ATT ACC | 132 | 56 | 35 |
|  | US | GGA TTA TTA ATA GTG TGT GGA GTG ATT TAT | 105 | 66 | 35 |
|  | UaS | CAC CTA ATA AAT TAC CAA CAC CCA CA | 105 | 66 | 35 |
|  | MS | TTA ATA GCG TGC GGA GTG ATT TAC | 90 | 66 | 35 |
|  | MaS | AAT TAC CGA CGC CCG CG | 90 | 66 | 35 |
| ***MGMT*** | FU | GYG TTT YGG ATA TGT TGG GAT AGT T | 135 | 56 | 35 |
|  | FD | AAA CTC CRC ACT CTT CCR AAA AC | 135 | 56 | 35 |
|  | US | TTT GTG TTT TGA TGT TTG TAG GTT TTT GT | 93 | 60 | 30 |
|  | UaS | AAC TCC ACA CTC TTC CAA AAA CAA AAC A | 93 | 60 | 30 |
|  | MS | TTT CGA CGT TCG TAG GTT TTC GC | 81 | 60 | 30 |
|  | MaS | GCA CTC TTC CGA AAA CGA AAC G | 81 | 60 | 30 |

***** *FU=flank up; FD=flank down; US=U sense; UaS=U anti sense; MS=M sense; MaS=M anti sense.*

**Supplementary figure S1: MSP analysis of NDRG1.**

*
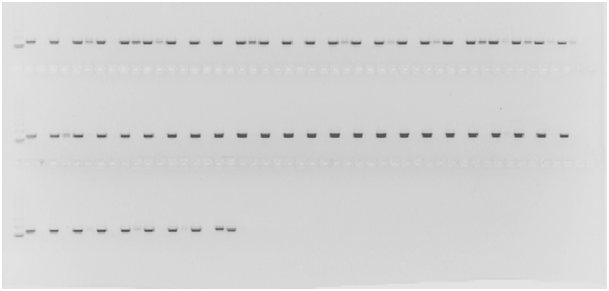
*

**Supplementary figure S2: MSP analysis of NDRG4.**

*
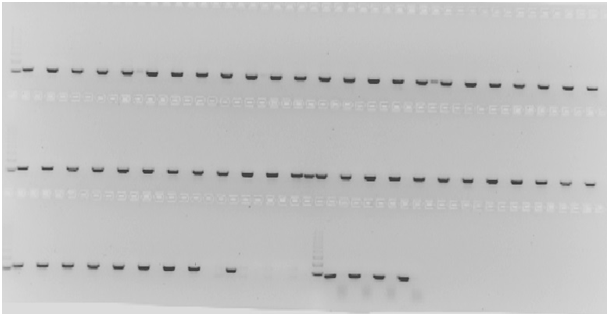
*
